# Notes and News

**Published:** 1906-06-02

**Authors:** 


### Notes and News.

The Hon. Rupert Guinness, C.M.G., will preside at the annual dinner of the Metropolitan Hospital on July 2 next at the Whitehall Rooms.

H.R.H. the Duke of Connaught will preside at the festival dinner of the Middlesex Hospital, to be held at the Mercers' Hall on June 12.

The Bishop of London, Mrs. Craigie, and Sir F. Treves will speak at the meeting of the Invalid Children's Aid Association on May 30, at 17 Bruton Street, W., at 3 o'clock.

H.R.H. Princess Louise, Duchess of Argyll, is interesting herself in a ball in aid of the funds of the Royal Waterloo Hospital on June 26 next at the Grafton Galleries, to which she has given her patronage.

